# Structure-guided multivalent nanobodies block SARS-CoV-2 infection and suppress mutational escape

**DOI:** 10.1126/science.abe6230

**Published:** 2021-01-12

**Authors:** Paul-Albert Koenig, Hrishikesh Das, Hejun Liu, Beate M. Kümmerer, Florian N. Gohr, Lea-Marie Jenster, Lisa D. J. Schiffelers, Yonas M. Tesfamariam, Miki Uchima, Jennifer D. Wuerth, Karl Gatterdam, Natalia Ruetalo, Maria H. Christensen, Caroline I. Fandrey, Sabine Normann, Jan M. P. Tödtmann, Steffen Pritzl, Leo Hanke, Jannik Boos, Meng Yuan, Xueyong Zhu, Jonathan L. Schmid-Burgk, Hiroki Kato, Michael Schindler, Ian A. Wilson, Matthias Geyer, Kerstin U. Ludwig, B. Martin Hällberg, Nicholas C. Wu, Florian I. Schmidt

**Affiliations:** 1 Core Facility Nanobodies, Medical Faculty, University of Bonn, 53127 Bonn, Germany.; 2 Institute of Innate Immunity, Medical Faculty, University of Bonn, 53127 Bonn, Germany.; 3 Department of Cell and Molecular Biology, Karolinska Institutet, 17177 Stockholm, Sweden.; 4 Department of Integrative Structural and Computational Biology, The Scripps Research Institute, La Jolla, CA 92037, USA.; 5 Institute of Virology, Medical Faculty, University of Bonn, 53127 Bonn, Germany.; 6 German Centre for Infection Research (DZIF), partner site Bonn-Cologne, 53127 Bonn, Germany.; 7 Institute of Structural Biology, Medical Faculty, University of Bonn, 53127 Bonn, Germany.; 8 Institute for Medical Virology and Epidemiology, Section Molecular Virology, University Hospital Tübingen, 72076 Tübingen, Germany.; 9 Department of Microbiology, Tumor and Cell Biology, Karolinska Institutet, 17177 Stockholm, Sweden.; 10 Institute of Human Genetics, Medical Faculty, University of Bonn, 53127 Bonn, Germany.; 11 Institute for Clinical Chemistry and Clinical Pharmacology, Medical Faculty, University of Bonn, 53127 Bonn, Germany.; 12 Institute of Cardiovascular Immunology, Medical Faculty, University of Bonn, 53127 Bonn, Germany.; 13 The Skaggs Institute for Chemical Biology, The Scripps Research Institute, La Jolla, CA 92037, USA.; 14 Centre for Structural Systems Biology (CSSB) and Karolinska Institutet VR-RÅC, Notkestrasse 85, 22607 Hamburg, Germany.; 15 Department of Biochemistry, University of Illinois at Urbana-Champaign, Urbana, IL 61801, USA.; 16 Carl R. Woese Institute for Genomic Biology, University of Illinois at Urbana-Champaign, Urbana, IL 61801, USA.; 17 Center for Biophysics and Quantitative Biology, University of Illinois at Urbana-Champaign, Urbana, IL 61801, USA.

## Abstract

The pandemic caused by severe acute respiratory syndrome coronavirus 2 (SARS-CoV-2) continues to spread, with devastating consequences. For passive immunization efforts, nanobodies have size and cost advantages over conventional antibodies. In this study, we generated four neutralizing nanobodies that target the receptor binding domain of the SARS-CoV-2 spike protein. We used x-ray crystallography and cryo–electron microscopy to define two distinct binding epitopes. On the basis of these structures, we engineered multivalent nanobodies with more than 100 times the neutralizing activity of monovalent nanobodies. Biparatopic nanobody fusions suppressed the emergence of escape mutants. Several nanobody constructs neutralized through receptor binding competition, whereas other monovalent and biparatopic nanobodies triggered aberrant activation of the spike fusion machinery. These premature conformational changes in the spike protein forestalled productive fusion and rendered the virions noninfectious.

## A double punch against SARS-CoV-2

Monoclonal antibodies are an important weapon in the battle against COVID-19. However, these large proteins are difficult to produce in the needed quantities and at low cost. Attention has turned to nanobodies, which are aptly named, single-domain antibodies that are easier to produce and have the potential to be administered by inhalation. Koenig *et al.* describe four nanobodies that bind to the severe acute respiratory syndrome coronavirus 2 (SARS-CoV-2) spike protein and prevent infection of cells (see the Perspective by Saelens and Schepens). Structures show that the nanobodies target two distinct epitopes on the SARS-CoV-2 spike protein. Multivalent nanobodies neutralize virus much more potently than single nanobodies, and multivalent nanobodies that bind two epitopes prevent the emergence of viral escape mutants.

*Science*, this issue p. eabe6230; see also p. 681

## Abstract (structured)

**INTRODUCTION:**

The global scale and rapid spread of severe acute respiratory syndrome coronavirus 2 (SARS-CoV-2) pose unprecedented challenges to society, health care systems, and science. In addition to effective and safe vaccines, passive immunization by antibody-related molecules offers an opportunity to harness the vertebrate immune system to fight viral infections in high-risk patients. Variable domains of heavy-chain–only antibodies (VHHs), also known as nanobodies, are suitable lead molecules in such efforts, as they are small, extremely stable, easy to engineer, and economic to produce in simple expression systems.

**RATIONALE:**

We engineered improved multivalent nanobodies neutralizing SARS-CoV-2 on the basis of two principles: (i) detailed structural information of their epitopes and binding modes to the viral spike protein and (ii) mechanistic insights into viral fusion with cellular membranes catalyzed by the spike.

**RESULTS:**

Nanobodies specific for the receptor binding domain (RBD) of SARS-CoV-2 spike were identified by phage display using nanobody libraries from an alpaca and a llama immunized with the RBD and inactivated virus. Four of the resulting nanobodies—VHHs E, U, V, and W—potently neutralize SARS-CoV-2 and SARS-CoV-2–pseudotyped vesicular stomatitis virus. X-ray crystallography revealed that the nanobodies bind to two distinct epitopes on the RBD, interfaces “E” and “UVW,” which can be synergistically targeted by combinations of nanobodies to inhibit infection. Cryo–electron microscopy (cryo-EM) of trimeric spike in complex with VHH E and VHH V revealed that VHH E stabilizes a conformation of the spike with all three RBDs in the “up” conformation (3-up), a state that is typically associated with activation by receptor binding. In line with this observation, we found that VHH E triggers the fusion activity of spike in the absence of the cognate receptor ACE2. VHH V, by contrast, stabilizes spike in a 2-up conformation and does not induce fusion. On the basis of the structural information, we designed bi- and trivalent nanobodies with improved neutralizing properties. VHH EEE most potently inhibited infection, did not activate fusion, and likely inactivated virions by outcompeting interaction of the virus with its receptor. Yet evolution experiments revealed emergence of escape mutants in the spike with single–amino acid changes that were completely insensitive to inhibition by VHH EEE. VHH VE also neutralized more efficiently than VHH E or VHH V alone; stabilized the 3-up conformation of spike, as determined by cryo-EM; and more strongly induced the spike fusogenic activity. We conclude that the premature activation of the fusion machinery on virions was an unexpected mechanism of neutralization, as enhanced neutralization could not be attributed simply to better blocking of virus-receptor interactions. Activation of spike in the absence of target membranes likely induces irreversible conformational changes to assume the energetically favorable postfusion conformation without catalyzing fusion per se. Simultaneous targeting of two independent epitopes by VHH VE largely prevented the emergence of resistant escape mutants in evolution experiments.

**CONCLUSION:**

Our results demonstrate the strength of the modular combination of nanobodies for neutralization. Premature activation of spike by nanobodies reveals an unusual mode of neutralization and yields insights into the mechanism of fusion.

## Teaser

SARS-CoV-2–neutralizing nanobodies were combined to design potent multivalent nanobodies.

## Graphical abstract

**Figure:**
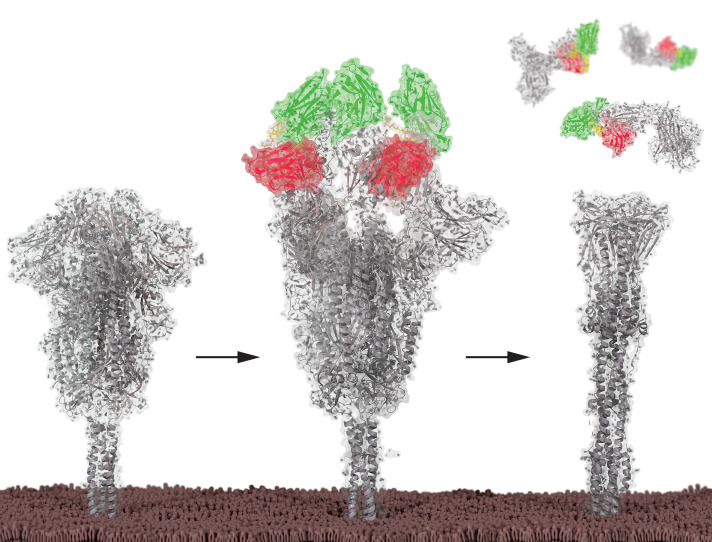
Bivalent nanobodies neutralize by inducing postfusion conformation of the SARS-CoV-2 spike. On virions, SARS-CoV-2 spike trimers are mostly in an inactive configuration with all RBDs in the down conformation (left). Binding of bivalent nanobody VE stabilizes the spike in an active conformation with all RBDs up (middle), triggering premature induction of the postfusion conformation, which irreversibly inactivates the spike protein (right).

The current pandemic caused by severe acute respiratory syndrome coronavirus 2 (SARS-CoV-2) poses serious challenges to patients, health care systems, and economic and social activity. Although efforts to develop vaccines are advancing rapidly, vaccines will likely not be suitable for immunocompromised patients. Additional therapeutic modalities for prophylaxis or treatment of high-risk patients are needed, as is testing of vaccines in children. Neutralizing antibodies or related molecules therefore offer great potential as immediate and direct-acting antiviral agents (*1*). Yet they cannot be easily and economically produced in sufficient amounts for mass application and cannot be readily modified to include multiple specificities without major costs in yield and quality. Conventional antibodies will also have to be assessed for any possibility of antibody-dependent enhancement (ADE) of infection (*2*). By contrast, variable domains of camelid heavy-chain–only antibodies (VHHs), also known as nanobodies, offer an opportunity to rapidly produce antiviral agents for passive immunization. Production in prokaryotic expression systems is cheap, is easily scaled up, and allows straightforward protein engineering, including multivalent nanobodies with enhanced functionalities (*3*). Nanobodies have favorable biochemical properties, including high thermostability and deep tissue penetration. ALX-0171, a trivalent nanobody that neutralizes respiratory syncytial virus, was developed for application using inhalators and accelerated viral clearance in patients, although treatment several days after symptom onset did not improve the clinical outcome (*4*).

The SARS-CoV-2 spike protein binds the cellular receptor ACE2 and catalyzes membrane fusion (*5*). Conformational flexibility of the trimeric spike protein allows each of its receptor binding domains (RBDs) to exist in two major configurations: a “down” conformation that is thought to be less accessible to binding of most neutralizing antibodies (NAbs) and an “up” conformation that binds ACE2 and most NAbs to the RBD (*6*–*8*). Many NAbs bind to the RBD of the spike protein and compete with ACE2 binding when the RBD is in the up conformation, thereby hindering infection (*9*, *10*). A few NAbs can bind to and stabilize the down conformation and thus prevent the conformational changes required for viral entry (*11*, *12*). Other mechanisms of neutralization or prevention of infection, such as antibody-dependent cellular toxicity (*13*), are possible, but none have been characterized in molecular detail.

## Camelid nanobodies that bind two different epitopes on the SARS-CoV-2 spike RBD neutralize infection

**Fig. 1:**
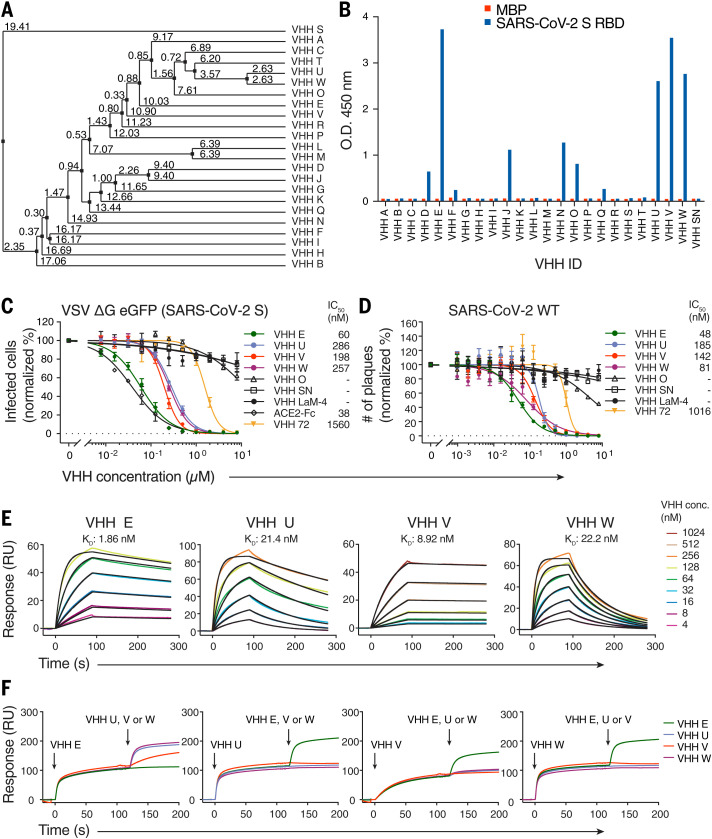
Camelid nanobodies against two epitopes on the SARS-CoV-2 spike RBD neutralize infection. (**A**) Average distance tree of nanobody candidates identified by phage display, calculated by percentage identity (*66*). (**B**) Binding of 100 nM HA-tagged VHHs to immobilized SARS-CoV-2 spike RBD or a control protein (MBP) was quantified by ELISA with horseradish peroxidase (HRP)–coupled anti-HA antibody. Unrelated VHH SN was used as a negative control. O.D., optical density. (**C**) SARS-CoV-2 spike–pseudotyped VSV ΔG eGFP was incubated with the indicated concentrations of HA- or LPETG-tagged (VHH LaM-4, VHH 72) VHHs or ACE2-Fc at 37°C for 1 hour, followed by infection of Vero E6 cells for 8 hours. eGFP-positive cells were measured by flow cytometry to quantify infection. Normalized values from three independent experiments ± SEM and IC_50_ values are plotted. (**D**) SARS-CoV-2 was incubated with the indicated concentrations of HA- or LPETG-tagged VHHs as in (C), followed by plaque assay on Vero E6 cells. Plaques were enumerated 3 days after infection; normalized values of three independent experiments ± SEM and IC_50_ values are plotted. (**E** and **F**) Biotinylated SARS-CoV-2 spike RBD was immobilized on SPR spectroscopy chips. (E) Indicated HA-tagged VHHs were injected for 90 s, followed by dissociation for 180 s. Dissociation constants (*K*_D_) were determined on the basis of fits, applying a 1:1 interaction model. (F) Epitope binning was performed by first injecting a single VHH for 120 s, followed by injection of a 1:1 mixture of the first nanobody in combination with VHH E, U, V, or W for 80 s.

We immunized one alpaca and one llama with the RBD of SARS-CoV-2 spike as well as formalin-inactivated SARS-CoV-2 to elicit neutralizing heavy-chain–only antibodies (fig. S1A). We identified 23 candidate nanobodies (Fig. 1A and fig. S1B) by phage display and confirmed binding of 10 hits by enzyme-linked immunosorbent assay (ELISA) (Fig. 1B and fig. S1, C and D). Their neutralizing activity was assessed in single-round infections with replication-deficient vesicular stomatitis virus (VSV) ΔG eGFP pseudotyped with SARS-CoV-2 spike Δ18. Four nanobodies—VHH E derived from the llama and VHHs U, V, and W from the alpaca—potently neutralized infection in a dose-dependent manner, with a half-maximal inhibitory concentration (IC_50_) value of 60 nM for the most potent nanobody, VHH E (Fig. 1C and fig. S1, E and F). The neutralizing activity of VHH E is thus similar to bivalent recombinant ACE2-Fc. Three nanobodies from a synthetic library did not neutralize (*14*), whereas neutralizing activity of nanobody Ty1 (*15*) was confirmed. As a monomer, SARS-CoV-1–specific nanobody VHH 72 (*16*) neutralized SARS-CoV-2–pseudotyped virus at concentrations above 1 μM (Fig. 1C) but potently inhibited SARS-CoV-1–pseudotyped virus at nanomolar concentrations (fig. S1G). None of the four nanobody hits neutralized SARS-CoV-1–pseudotyped virus (fig. S1H). Plaque reduction neutralization tests (PRNTs) with SARS-CoV-2 on Vero E6 cells confirmed the neutralizing activity, yielding IC_50_ values ranging from 48 to 185 nM (Fig. 1D). We further validated virus neutralization microscopically by quantifying the replication of an mNeonGreen-expressing clone of SARS-CoV-2 on human Caco2 cells in the presence of nanobodies over time (fig. S1I and movies S1 to S7). We measured binding affinities of the VHHs to the RBD by surface plasmon resonance (SPR) (Fig. 1E and fig. S1J) and obtained dissociation constants of 2 nM (VHH E), 21 nM (VHH U), 9 nM (VHH V), and 22 nM (VHH W) in the kinetic mode (table S1). An SPR-based binding competition assay revealed that the nanobodies bind to two distinct regions: U, V, and W competed for the same binding interface on RBD (interface *UVW*), whereas VHH E binds to a different RBD interface (interface *E*) and could bind to the RBD at the same time as U, V, or W (Fig. 1F).

## Binding epitopes of neutralizing VHHs on the spike RBD

**Fig. 2:**
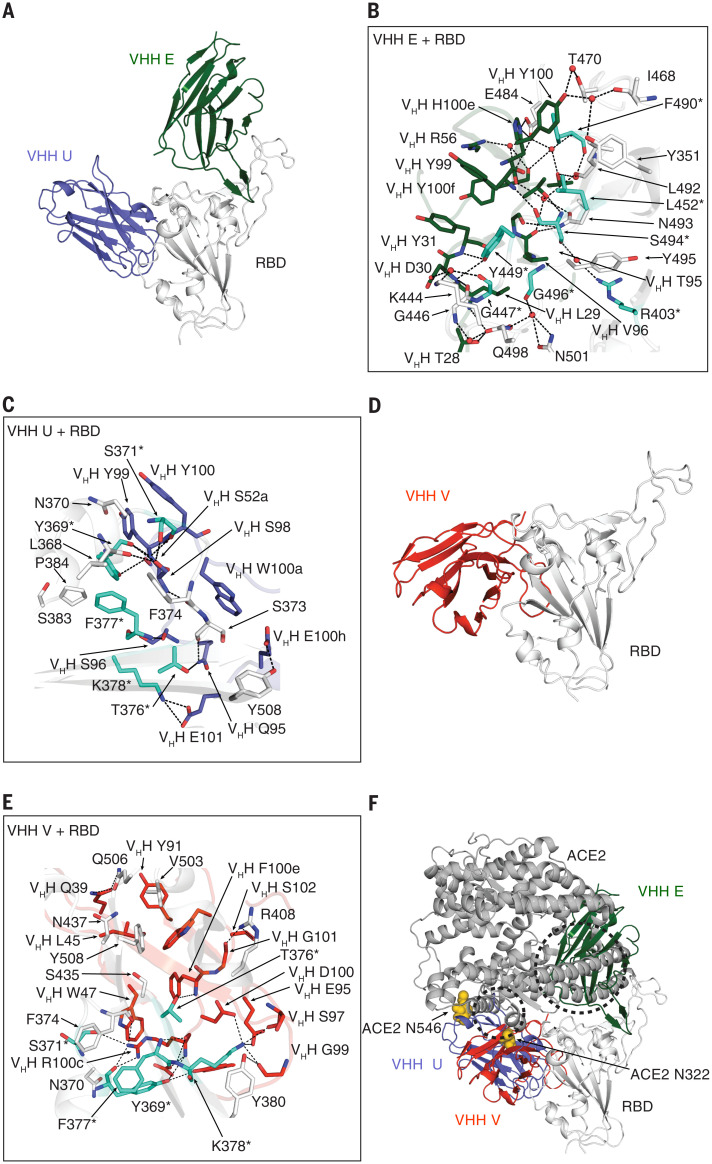
X-ray crystallography defines the binding sites of neutralizing VHHs on the SARS-CoV-2 RBD. (**A** to **C**) Crystal structure of SARS-CoV-2 spike RBD in complex with VHH E and VHH U at 1.87 Å (A) and detailed interaction interface of RBD (in white) with VHH E (B) and RBD with VHH U (C), respectively. (**D** and **E**) Crystal structure of SARS-CoV-2 spike RBD in complex with VHH V at 2.55 Å (D) and detailed interaction interface of RBD with VHH V (E). Escape mutants (see Fig. 5 and tables S5 to S8) in the RBD are highlighted in teal and labeled with asterisks. (**F**) Overview of binding sites of three neutralizing nanobodies on the RBD and their overlap with ACE2, based on PDB ID 6M0J (*67*). Steric clashes with VHH E are indicated within the dashed circles. N-glycans at N322 and N546 of ACE2 are depicted as yellow spheres. All structural analyses of VHH U and VHH E in complex with RBD were based on one of the two copies in the asymmetric unit with closer alignment to the localized reconstructions of VHH E with RBD and VHH VE with RBD using cryo-EM. Single-letter abbreviations for the amino acid residues are as follows: A, Ala; C, Cys; D, Asp; E, Glu; F, Phe; G, Gly; H, His; I, Ile; K, Lys; L, Leu; M, Met; N, Asn; P, Pro; Q, Gln; R, Arg; S, Ser; T, Thr; V, Val; W, Trp; and Y, Tyr.

We next determined x-ray crystal structures of complexes of VHH E and VHH U with SARS-CoV-2 RBD at 1.87 Å (Fig. 2, A to C), VHH V with RBD and Fab CC12.3 (*9*) at 2.55 Å (Fig. 2, D and E), and VHH W with RBD and Fab CC12.3 at 2.73 Å (fig. S2, A and B) (table S2). VHH E and U bind to distinct epitopes on the RBD (Fig. 2A). VHH E employs its complementarity determining region (CDR) 1 and its unusually long CDR3 (22 amino acids) to bind the ACE2 binding site on the RBD (Fig. 2B). The VHH E epitope is similar to that of the potent SARS-CoV-2–neutralizing antibody CC12.3 (*17*) and nanobodies H11-D4 (*18*), MR17, and SR4 (*19*) (fig. S2C). Nevertheless, VHH E binds in an orientation that markedly differs from that of other neutralizing nanobodies (fig. S2C). Its CDR3 adopts an extended β-hairpin conformation that inserts into the receptor binding site (RBS) using both polar and hydrophobic interactions as well as bridging water molecules (Fig. 2B). The flexibility of CDR3 is confined at one end by a disulfide bond between C50 in CDR2 and C100h (Kabat numbering) in CDR3 that is involved in a hydrophobic and aromatic patch that interacts with the RBS (fig. S2H). Of the 27 epitope residues that bind VHH E, 16 are also involved in ACE2 binding (Fig. 2B) (*20*).

Although VHH U binds to a distinct epitope on the RBD, it should also compete with ACE2 for binding to the RBD, owing to clashes with the ACE2 protein and with the glycans at N322 and N546 of ACE2 (Fig. 2F and fig. S2D). VHH U uses all three CDRs to bind the RBD. CDR3 is tethered to CDR2 by a disulfide bond between C100b and C50 that creates two distinct loops in a flat, bilobed CDR3 that interact with the RBD (fig. S2H). The epitope of VHH U overlaps with the binding site of SARS-CoV-1–neutralizing antibody CR3022 (*17*) and VHH 72 (*16*) (fig. S2C). Although most of the residues involved in the interaction of the SARS-CoV-2 RBD with VHH U are identical in the SARS-CoV-1 RBD, VHH U does not neutralize SARS-CoV-1 (fig. S1H). In agreement with the neutralization data, fluorescently labeled VHH U binds to SARS-CoV-2 spike transiently expressed on human embryonic kidney (HEK) 293T cells, but not to SARS-CoV-1 spike (fig. S2G). SARS-CoV-1 spike N357 is part of an NxT sequon for N-glycosylation, whereas the corresponding SARS-CoV-2 N370 is not glycosylated. Binding of VHH U to SARS-CoV-1 spike is partly restored when T359 is mutated to alanine and the sequon is eliminated, suggesting that differential glycosylation contributes to different binding properties (fig. S2G). Binding of VHH E and U does not substantially alter the overall RBD fold [Cα root mean square deviation (RMSD) = 0.37 Å between RBDs bound by E and U, and ACE2].

VHH W adopts a structure similar to that of VHH U (Cα RMSD = 0.48 Å) and engages the RBD in the same way (Fig. 2A and fig. S2, A and F), consistent with the finding that six of the seven amino acid differences between the two nanobodies occur in the framework and are not involved in binding. Although VHH V binds to a similar epitope as that bound by VHH U and W, it is oriented differently on the RBD. In this case, CDR3 is mainly involved in the binding, and no major changes in the overall RBD structure were observed upon VHH V binding. Binding of VHH V is also expected to compete with ACE2 binding, owing to a steric clash with the glycan at N322 and potentially at N546 of ACE2 (Fig. 2F and fig. S2D). To confirm that nanobody binding to the RBD competes with ACE2 binding on the plasma membrane, we performed a flow cytometry–based competition assay and quantified binding of fluorescently labeled RBD to ACE2-expressing HEK 293T cells (fig. S2E). ACE2 binding was indeed outcompeted by all neutralizing VHHs in a dose-dependent manner, with VHH V being slightly less potent than the others.

## Neutralizing nanobodies stabilize the SARS-CoV-2 spike in the RBD-up conformation

**Fig. 3:**
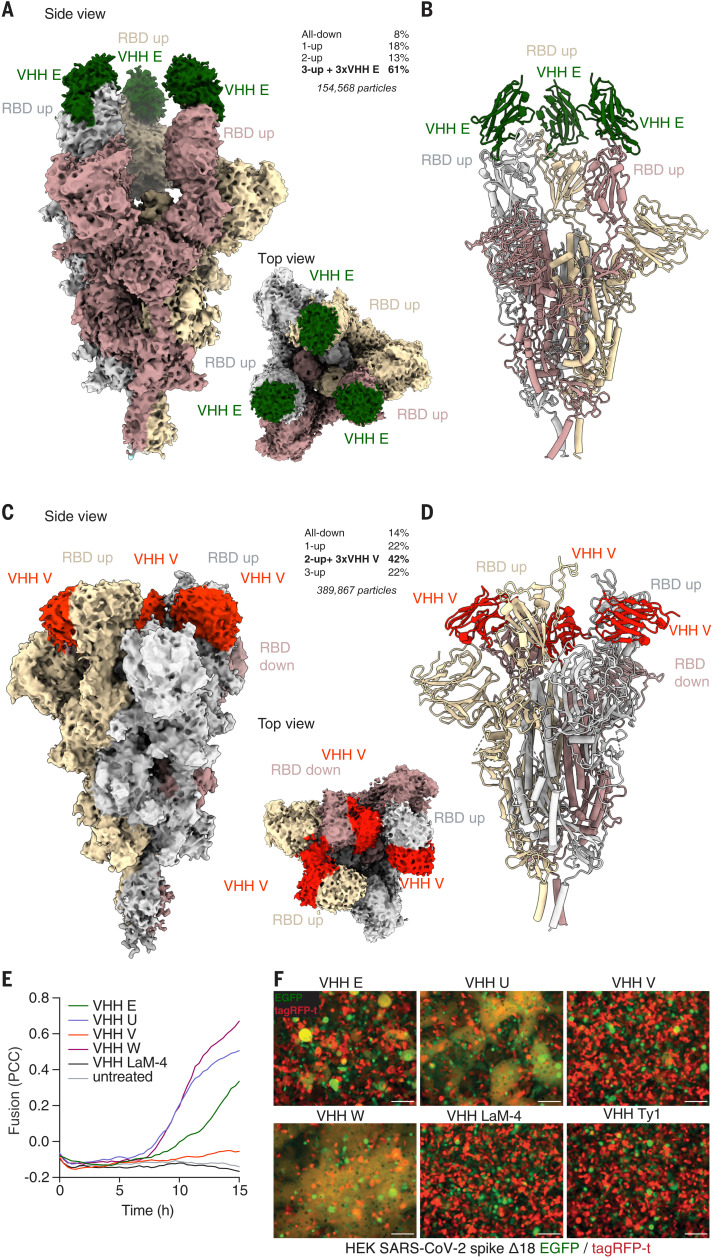
Cryo-EM structures reveal that VHHs stabilize SARS-CoV-2 spike trimers with RBDs in the up conformation. (**A** to **D**) Cryo-EM reconstructions [(A) and (C)] and atomic models [(B) and (D)] of VHH E [(A) and (B)] and VHH V [(C) and (D)] in complex with trimeric SARS-CoV-2 spike. Frequencies of the identified complexes as well as total numbers of considered particles are noted. (A and B) VHH E (in green) binds to SARS-CoV-2 in a 3-up conformation in the most abundant complex; the resolution is 3.3 Å [0.143 Fourier shell correlation (FSC)]. (C and D) VHH V (in red) binds to SARS-CoV-2 in a 2-up conformation with all VHH binding sites occupied at a resolution of 3.0 Å (0.143 FSC). In the most abundant complex, VHH V binds to the RBD in the up or the down conformation. (**E** and **F**) HEK 293 cells inducibly expressing SARS-CoV-2 S Δ18 and either eGFP or tagRFP-t were seeded into microscopy-grade 96-well plates in a 1:1 ratio and induced with 1 μg/ml doxycycline for 20 hours. Cells were treated with 1 μM of the indicated VHHs, and microscopy images were recorded every 20 min for 14 hours at 37°C. (E) Fusion was quantified by calculating Pearson correlation coefficients (PCC) between eGFP and tagRFP-t. Average values from four fields of view of an experiment representative of three independent experiments are displayed. (F) Representative images of cells 12 hours after treatment are displayed (also see fig. S13 and movies S8 to S13). Scale bars, 100 μm.

The RBDs exist in an equilibrium of up and down conformations in the context of the trimeric spike on virions. Most unperturbed spike trimers exist in the configuration with no or one RBD up, whereas the form with all three RBDs in the up conformation (3-up) is barely populated (*6*, *7*, *21*). Only the up conformation of the RBD is compatible with ACE2 (*22*) binding, which likely induces further conformational changes that favor secondary proteolytic cleavage, dissociation of the S1 subdomain, and eventually conversion to the postfusion conformation (*23*). However, it is unknown how many RBDs must be in the up conformation to permit each of these transitions. To further investigate the mechanism of action of the identified neutralizing nanobodies, we used cryo–electron microscopy (cryo-EM) to determine structures of the soluble, trimeric spike complex bound to individual nanobodies (*6*, *24*). To stabilize the prefusion conformation, mutants of spike lacking the furin cleavage site and containing stabilizing proline substitutions were used throughout this study (*6*). Our initial analysis focused on one representative nanobody for each of the two identified epitopes. Ab initio reconstruction of VHH E bound to the trimeric spike revealed that the predominant complex (61%) contained all RBDs in the up conformation (3-up) with all three VHH E binding sites populated (Fig. 3, A and B, and figs. S3 to S5). The remaining particles resulted in very-low-resolution structures without any density for VHH E. Spike structures containing one or more RBDs in the down conformation were not compatible with binding of three molecules of VHH E, as substantial clashes with the spike glycans or between VHHs were observed when the VHH E structures were modeled into different spike quaternary structures (fig. S6). This finding suggests that binding of VHH E stabilizes (or induces) the 3-up conformation by trapping the RBD in the up-state. Once bound to VHH E, the RBD is unable to access the down conformation.

The cryo-EM structure of trimeric spike in complex with VHH V revealed that the predominant complex (42% of particles) had all three RBDs bound by VHH V (Fig. 3, C and D, and figs. S7 to S9), with two RBDs in the up conformation and one in the down conformation. To accommodate three molecules of VHH V, one of the RBDs in the up conformation was displaced by 8 Å, and the RBD in the down conformation was displaced by 11 Å, relative to the 2-up, 1-down conformation without nanobodies (*25*) (figs. S10 and S11). The remaining complexes did not contain VHH V. Apart from the RBD state, the overall structure of the trimeric spike was not substantially changed by any of the nanobodies. However, VHH E and VHH V altered the abundance of spike-trimer conformational states, and the cryo-EM structures confirmed the binding modes as identified by macromolecular crystallography. Molecular modeling of the VHH V–RBD interface revealed no differences to the crystallographic structure. A small backbone difference in RBD residues 446 to 451 in the interface with VHH E was noted, albeit in different resolution structures and in different complexes (fig. S12A).

## Neutralizing nanobodies trigger activation of the fusion machinery

ACE2 can only bind to RBDs in the up conformation and is expected to trigger conformational changes required for secondary proteolytic processing and fusion. Premature activation of these steps will inactivate the fusion machinery, as the energetically favored postfusion conformation is irreversible. To test whether stabilization of the RBDs in the up conformation by nanobodies activates the fusion machinery, we generated a HEK 293–based cell line that can be induced to express the SARS-CoV-2 spike protein on the cell surface. We further generated derivative cell lines that additionally and constitutively express either eGFP or tagRFP-t. Because HEK 293 cells do not express the cognate receptor ACE2, the SARS-CoV-2 spike is not expected to mediate cell-cell fusion. We mixed the green and red fluorescent cell lines at an equal ratio, treated them with nanobodies, and recorded cells by fluorescence microscopy during a period of 14 hours (Fig. 3, E and F, and fig. S13, A and B). Cell-cell fusion, as a macroscopic readout for activation of the viral spike protein, would result in cells containing both eGFP and tagRFP-t. Untreated cells, or cells treated with control nanobodies, did not fuse or exhibit any signs of toxicity. However, incubating spike-expressing cells with VHH E, U, or W resulted in cell-cell fusion, as quantified by mixed eGFP and tagRFP-t fluorescence (Fig. 3F). VHH-induced fusion was particularly prominent for VHH U and W, resulting in fusion of all cells in the field of view, and was somewhat weaker for VHH E. VHH V barely induced fusion, whereas no fusion was observed in the presence of VHH Ty1. Stabilization of the RBD up conformation by neutralizing nanobodies may thus favor conformational changes that mediate fusion. Furthermore, we analyzed fusion between ACE2- and SARS-CoV-2 spike–expressing cell lines (fig. S14). Upon induction of spike expression, near complete cell-cell fusion was observed within 12 hours. When expression was induced in the presence of VHH E, spike-ACE2–mediated fusion was reduced to 50%, consistent with VHH E binding to the ACE2 binding site. No inhibition was observed in the presence of control nanobodies or in the presence of VHHs U, V, W, or Ty1. This fusion assay therefore recapitulated the results of the infection-based neutralization assays.

It remains unclear whether nanobody binding is sufficient to induce fusion directly or whether it rather facilitates secondary proteolytic processing of spike as necessary for fusion. VHH E–induced fusion was partly inhibited by a protease inhibitor cocktail (fig. S13), suggesting that extracellular or membrane-associated proteases may be involved in the VHH-triggered fusion, as described for ACE2-triggered fusion by SARS-CoV-1 spike (*26*). It is thus possible that VHH binding mimics binding of ACE2 to the spike protein and exposes otherwise inaccessible protease cleavage sites. Regardless of the exact mechanism, only neutralizing nanobodies were found to trigger spike-mediated cell-cell fusion, suggesting that aberrant activation of the fusion machinery is involved in the mechanism of neutralization.

## Multivalent VHH fusions potentiate neutralization of SARS-CoV-2

**Fig. 4:**
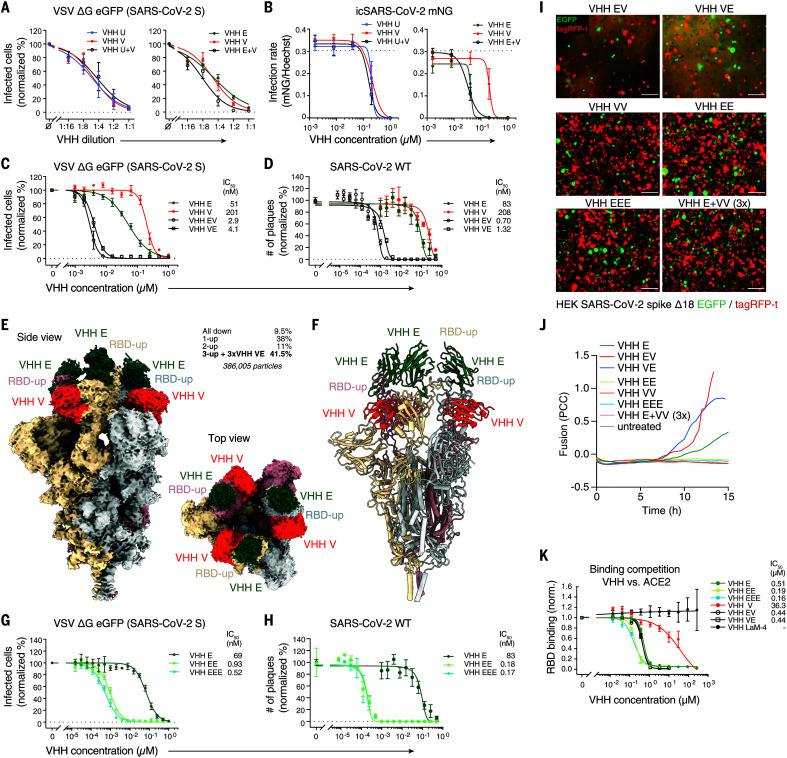
Multivalent VHH fusions potentiate neutralization of SARS-CoV-2. (**A**) SARS-CoV-2 spike–pseudotyped VSV ΔG eGFP was incubated with twofold serial dilutions of 0.25 μM VHH E, 1 μM VHH U, 1 μM VHH V, or the indicated combinations containing 50% of each VHH at 37°C for 1 hour. Vero E6 cells were subsequently incubated with the mixtures, and infection was quantified as in Fig. 1B. Normalized values from three independent experiments ± SEM are plotted. (**B**) Caco-2 cells were infected with mNeonGreen-expressing infectious-clone-derived SARS-CoV-2 (icSARS-CoV-2-mNG) in the presence of the indicated nanobody concentrations. Cells were fixed 48 hours postinfection and stained for DNA, and infection was quantified by microscopy. Normalized values from three independent experiments ± SEM are plotted. (**C** and **G**) SARS-CoV-2 spike–pseudotyped VSV ΔG eGFP was incubated with the indicated concentrations of HA-tagged single, bivalent, or trivalent VHHs at 37°C for 1 hour, followed by infection of Vero E6 cells as in Fig. 1C. Normalized values from three independent experiments ± SEM and IC_50_ values are plotted. (**D** and **H**) SARS-CoV-2 was incubated with the indicated concentrations of HA-tagged VHHs, followed by plaque assay on Vero E6 cells as in Fig. 1D. Normalized values of three independent experiments ± SEM and IC_50_ values are plotted. (**E** and **F**) Cryo-EM reconstruction (E) and atomic models (F) of VHH VE in complex with trimeric SARS-CoV-2 spike. Frequencies of the identified complexes as well as total number of considered particles are noted. The biparatopic VHH binds to SARS-CoV-2 in the 3-up conformation; the resolution is 2.62 Å (0.143 FSC). (**I** and **J**) HEK 293 cell lines inducibly expressing SARS-CoV-2 S Δ18 and either eGFP or tagRFP-t were treated with the indicated VHHs and analyzed as in Fig. 3, E and F, displaying representative images after 12 hours (I), as well as quantified fusion (J) (also see fig. S22 and movies S14 to S22). Scale bars, 100 μm. (**K**) HEK 293T cells expressing ACE2-tagRFP-t were incubated with DyLight 488–labeled SARS-CoV-2 spike RBD in the presence of the indicated concentrations of nanobodies. RBD bound to ACE2-positive cells was quantified by flow cytometry. Normalized data from three independent experiments ± SEM are plotted. Data presented in fig. S2E and Fig. 4K are from the same experiments, and values for VHHs E, V, and LaM-4 in fig. S2E are plotted for reference.

In the course of the natural adaptive immune response, viruses face a polyclonal antibody response, which may enhance neutralizing activity by additive or synergistic effects (*27*). To test whether the four neutralizing nanobodies act synergistically, we compared the neutralizing activity of combinations of individual nanobodies. For direct comparison, we established starting concentrations of the individual nanobodies that led to similar dose-response curves in a twofold dilution series (Fig. 4A and fig. S15A). We then compared neutralizing activities of the individual nanobodies with mixtures of nanobodies containing 50% of the concentration of each nanobody combined. Combinations of nanobodies competing for the same epitope (VHHs U and V, U and W, or V and W) exhibited additive inhibition—i.e., mixtures containing half of the concentrations of two nanobodies neutralized to the same extent as did the individual nanobodies alone at full concentration (Fig. 4A and fig. S15A). However, when nanobodies binding to different epitopes were combined (VHHs E and U, E and V, or E and W), mixtures containing 50% of the two VHHs neutralized more efficiently than 100% of each of the individual nanobodies. Combinations of nanobodies that bound to independent epitopes were also more potent in preventing SARS-CoV-2 mNeonGreen replication in human cells (Fig. 4B and fig. S15B).

These results suggest that targeting multiple epitopes may be beneficial to interfere with infection. Thus, we produced multivalent nanobody fusions linked by flexible linkers, capitalizing on the relatively unrestrained N and C termini of VHHs. On the basis of a superimposition of complex structures VHH E and VHH V binding to the same RBD, we determined that the C terminus of VHH V and the N terminus of VHH E were close enough (33 Å) to allow fusion with a 15–amino acid–long (GGGGS)_3_ linker (VHH VE) (*28*). The distance between the C terminus of VHH E and the N terminus of VHH V, by contrast, was estimated to be greater than the length of a 15–amino acid linker. The reverse construct (VHH EV) with a (GGGGS)_3_ linker was thus expected to bind to two different RBDs within a trimeric complex, to employ only one of its binding sites, or to induce some strain or distortion on the targeted RBD. The biparatopic nanobodies VHH VE and EV were produced in bacteria, purified, and subsequently subjected to SPR analysis (fig. S15C). With apparent dissociation constants of 84 pM (VE) and 200 pM (EV), the binding strength of the biparatopic nanobodies to the RBD was, respectively, 22 and 9 times that of the best monoparatopic VHH (VHH E). Although we also confirmed binding to trimeric spike (fig. S15D), the complex binding behavior precluded the application of standard affinity-calculation models. In neutralization assays with SARS-CoV-2 S–pseudotyped VSV, we found that VE or EV neutralized with IC_50_ values of 4.1 or 2.9 nM—i.e., 12 or 18 times more effective than the neutralizing activity of VHH E alone—respectively (Fig. 4C). We observed similar values for the biparatopic fusions VHH EW and WE (fig. S15E). Neutralizing activity of VHH fusions, as measured by PRNTs with wild-type (WT) SARS-CoV-2, was improved even more, attaining IC_50_ values of 0.7 nM for EV and 1.32 nM for VE (Fig. 4D).

Through examination of the cryo-EM reconstructions of VE in complex with trimeric spike (Fig. 4, E and F, and figs. S16 and S17), we observed that the biparatopic VHH VE stabilized all RBDs in the up conformation, and all six VHH binding sites were occupied on the spike. This configuration was found in 41.5% of all complexes; the remaining spike complexes did not contain any VHH (fig. S18). Although the linker itself was not visible, the 15–amino acid linker length between VHHs V and E is compatible with the observed distance between the C terminus of VHH V and the N terminus of VHH E on the same RBD (35 Å) (*28*), but not with the distance between VHHs V and E on different RBDs (>80 Å) (fig. S19). Through localized reconstruction techniques, we were able to obtain a map of the VHH VE–RBD region of the full spike that enabled molecular modeling. Notably, the small difference in the backbone around Y449 observed in the localized reconstruction of the VHH E–RBD structure now corresponded in VHH VE–RBD to the conformation seen in the high-resolution x-ray structure of RBD VHH E and VHH U (fig. S12B). These differences hint at possible allostery between the two epitopes, but it is not feasible to determine this with certainty, given the resolution of the present EM reconstructions in the two areas. Complexes of the spike trimer with VHH V alone (Fig. 3, C and D) contained two RBDs in the up conformation and one in the down conformation. Hence, the formation of a 3-up spike trimer is dependent on the inclusion of VHH E in the biparatopic VHH VE complex (fig. S20).

The cryo-EM structures of VHH E in complex with the trimeric spike suggested that three RBDs in the up conformation could also be bound by multivalent fusions of VHH E. We thus produced VHH EE and VHH EEE connected by GS-rich linkers of 15–amino acid length and similarly tested their neutralizing activity. VHH EE and VHH EEE neutralized VSV ΔG eGFP (SARS-CoV-2 S) with IC_50_ values in the pM range (930 and 520 pM, respectively) (Fig. 4G). IC_50_ values in PRNTs with WT virus were even lower, reaching 180 and 170 pM for VHH EE and EEE, respectively (Fig. 4H). We also tested VHH VV and found that it binds RBD with higher apparent affinity than does VHH V alone (fig. S15C). Yet VHH VV improved neutralizing activity only modestly (fig. S15F), possibly because VHH VV is not expected to simultaneously bind different RBDs within the trimeric spike.

## Monovalent, multivalent, and biparatopic nanobodies boost SARS-CoV-2 neutralization by distinct mechanisms

Next, we explored the mechanism by which multivalent nanobodies enhance the neutralizing activity of individual nanobodies. We first tested whether multivalent nanobodies outcompeted binding of fluorescently labeled RBD to ACE2-expressing HEK 293T cells (Fig. 4K). VHHs EE and EEE reduced binding to RBD better than VHH E alone, suggesting that the increased (apparent) affinity of these nanobodies may better prevent virus binding to receptor than VHH E. Although VHHs EE and EEE exhibit a threefold lower IC_50_ than VHH E in the RBD competition assays, it is possible that other factors contribute to the 100-fold enhanced neutralizing activity. VHHs VE and EV did not compete with RBD-ACE2 interactions substantially better than VHH E alone. Hence, we conclude that their improved neutralizing activity must be explained by other mechanisms.

We next tested whether incubation of SARS-CoV-2 spike–expressing cells with multivalent nanobodies triggers spike-mediated cell-cell fusion in the absence of ACE2, as observed for VHHs E, U, and W (Fig. 4, I and J). VHHs EV and VE caused extensive cell-cell fusion, clearly exceeding the fusogenic activity of the individual nanobodies alone. VHHs EW and WE caused a similar degree of cell-cell fusion. We conclude that binding of VHHs EV, VE, EW, and WE to the spike potently induces conformational changes required for fusion. Moreover, the enhanced fusogenic activity of EV and VE correlates with the improved neutralizing activity over VHH E, which could not be explained by ACE2 competition. In VHH-mediated cell-cell fusion assays, cells were treated with nanobodies one day after induction of spike expression, exposing a large number of spike trimers at the cell surface at the onset of the experiment, including sites in close proximity to membranes of neighboring cells. Activation of spikes likely catalyzed cell-cell fusion. During neutralization of virions, however, nanobodies bind to spike on virions in the absence of target membranes. Thus, premature activation of this metastable state cannot facilitate infection but rather irreversibly converts the spike into the postfusion conformation precluding bona fide fusion events upon host cell contact.

The homobivalent and homotrivalent VHHs EE and EEE did not induce fusion despite a substantial boost in neutralizing activity, suggesting that binding of multiple covalently coupled nanobodies to the same epitope is not compatible with induction of fusion. To test whether cross-linking of individual spike promoters itself interferes with fusion, we incubated spike-expressing cells with fusogenic VHH U and a threefold molar excess of VHH EEE (fig. S22). Yet VHH U–mediated fusion was not restricted by VHH EEE. Fusion induced by VHH E, however, was inhibited by bivalent VHH VV, demonstrating that VHH binding can interfere with fusion in other ways, perhaps by preventing 3-up conformations.

In addition, we tested whether multivalent nanobodies interfered with fusion of SARS-CoV-2 spike– and ACE2-expressing cells. We induced spike expression at the onset of the experiment; therefore, newly synthesized spike that arrived at the plasma membrane faced an excess of nanobodies. Depending on the neutralization potential and the concentration of nanobodies, this may induce premature activation of spike by nanobodies or may interfere with binding of spike to ACE2, recapitulating the setup of neutralization assays. In line with the previously determined IC_50_ values, we found that fusion was partly inhibited by VHHs EE, EEE, EV, and VE (fig. S21). VHH VV did not inhibit fusion, whereas VHHs EW and WE had intermediate phenotypes. ACE2-mediated fusion assays therefore demonstrated that only the most potent neutralizing nanobodies interfered with spike-ACE2–triggered fusion at the studied concentration.

## Targeting two independent vulnerable epitopes on the RBD prevents viral escape

**Fig. 5:**
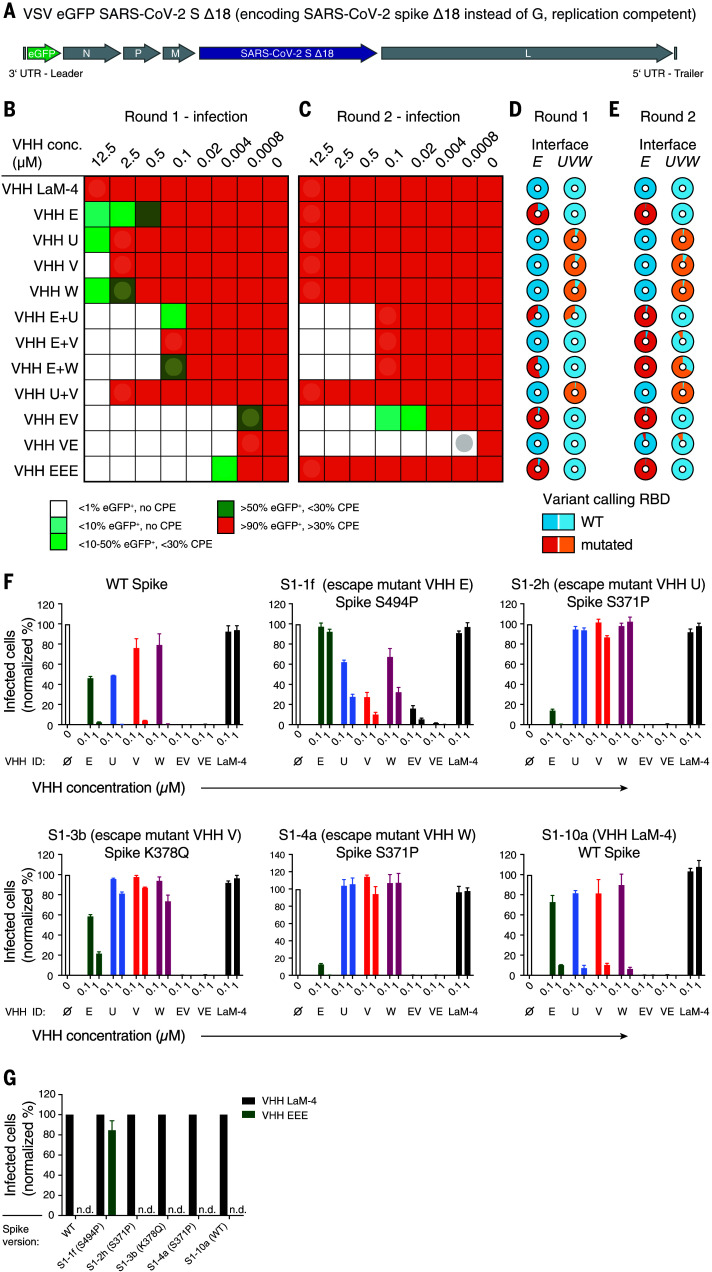
Simultaneous targeting of two independent epitopes with neutralizing VHHs prevents viral escape. (**A**) Genome structure of VSV SARS-CoV-2 S Δ18 eGFP. UTR, untranslated region. (**B** to **E**) Evolution experiment. (B) Replication-competent VSV SARS-CoV-2 S Δ18 eGFP at a multiplicity of infection (MOI) of 0.5 was incubated with different concentrations of the indicated VHHs and allowed to replicate on Vero E6 cells in 12 wells for 4 days. The fraction of infected (eGFP-positive) cells and the cytopathic effect (CPE) were estimated microscopically and are plotted according to the indicated color code. (C) Cleared supernatants from the wells indicated with a circle in (B) were diluted with four volumes of fresh infection medium (1:5 dilution) and used for a second round of replication on Vero E6 cells in the presence of the indicated VHH concentrations. Cleared supernatants were harvested as in (B). (D and E) Cell lysates from the wells indicated by circles in (B) and (C) [corresponding to (D) and (E), respectively] were subjected to targeted resequencing of the RBD to identify variants that had emerged at the interfaces to VHH E (interface *E*) or to VHH U, V, or W (interface *UVW*) and to quantify their allelic distribution (see tables S5 and S6 for details on detected variants). (**F** and **G**) Wild-type VSV SARS-CoV-2 spike eGFP or plaque-purified escape mutants of VHH E (S1-1f, Spike S494P), VHH U (S1-2h, Spike S371P), VHH V (S1-3b, Spike K378Q), VHH W (S1-4a, Spike S371P), and VHH LaM-4 (S1-10a, WT spike) at an MOI of 0.5 were incubated with the indicated VHH concentrations (F) or 1 μM of the indicated VHH (G) and used for Vero E6 infection experiments as in Fig. 1B. Infection was quantified by flow cytometry; normalized data from three independent experiments ± SEM are plotted. n.d., not detected.

In the course of prolonged infections or therapeutic settings, viruses with escape mutants emerge that evade recognition, and thus neutralization, by specific antibodies. We experimentally tested whether combinations of nanobodies targeting distinct epitopes, or our biparatopic nanobodies, increased resistance to escape mutants (see extended results in the supplementary materials). Briefly, we generated a chimeric, replication-competent VSV strain that expresses eGFP and encodes SARS-CoV-2 spike Δ18 instead of its own glycoprotein (VSV eGFP SARS-CoV-2 S; see Fig. 5A) (*29*–*31*). Vero E6 cells were infected with the chimeric virus in the presence of increasing concentrations of nanobodies or nanobody combinations and cultivated for 4 days (Fig. 5B). Virus from the wells with the highest nanobody concentration that still allowed replication (>10% infected cells) was collected and used for a second round of selection (Fig. 5C). Viral RNAs were extracted from infected cells after both rounds of selection and analyzed by next-generation sequencing (NGS) to identify emerging variants (Fig. 5, D and E). No mutations occurred in the presence of control nanobody VHH LaM-4. In the presence of VHH E or VHHs U, V, and W, replication at neutralizing concentrations was observed in the first round, and more than 85% of all viral sequences exhibited mutations in the structurally defined interfaces *E* or *UVW*, respectively. Replication in the second round was seen at all nanobody concentrations, and nearly all sequences contained nonsynonymous mutations in the nanobody epitope, indicating rapid selection for escape mutants. Particularly prominent were escape mutations F490S or S494P in the VHH E interface, as well as S371P and K378Q in the *UVW* interface, which are expected to compromise the structurally defined VHH–RBD interface (see extended results in the supplementary materials). We identified further mutations in interfaces *E* (G447S, Y449H/D/N, L452R, F490S, S494P/S, G496S, and Y508H) and *UVW* (Y369H, S371P, F374I/V, T376I, F377L, and K378Q/N) in this and a separate evolution experiment (tables S5 to S8).

Individual escape mutants encoding spike mutants S494P (VHH E), S371P (VHH U/W), and K378Q (VHH V) were plaque purified and thoroughly characterized (fig. S23, A and B). As expected, spike S494 mutants were resistant to VHH E and VHH EEE, whereas spike mutants S371P and K378Q were resistant to VHHs U, V, and W (Fig. 5, F and G). Escape mutants resistant to one VHH were systematically more sensitive to VHHs EV and VE than to a single nanobody that could still bind to their RBD. This finding suggests that residual binding to the mutated interface may still contribute to neutralization. Ectopically expressed mutants of SARS-CoV-2 spike were no longer stained by fluorescently labeled VHHs targeting the affected epitope, indicating that the detected mutations alone explained the loss of sensitivity (fig. S23C).

Combinations of VHHs E and U, E and V, and E and W did not allow the emergence of escape mutants that were resistant to both nanobodies in the second round of selection (Fig. 5C). Nevertheless, virus with mutations in at least one interface was enriched, and sequences containing mutations in both interfaces were detected after two rounds of replication in the presence of VHHs E and W (Fig. 5E). Replication in the presence of VHH EEE or combinations of VHHs U and V allowed the rapid emergence of escape mutations with nearly completely mutated interfaces in the first round of selection (Fig. 5D). Emerging virus was consequently able to replicate at all nanobody concentrations in the second round (Fig. 5C).

No virus resistant to biparatopic VHHs EV and VE emerged during two rounds of selection (Fig. 5C). Although VHH VE did not lead to the accumulation of any mutations, VHH EV selected for virus mutated in the *E* interface (Fig. 5E). The evolution experiments clearly demonstrate that simultaneous targeting of two neutralizing epitopes severely hampers or prevents the emergence of escape mutants. A single-point mutant was sufficient to escape the most potent homotrivalent nanobody, VHH EEE.

## Discussion

Nanobodies represent a versatile alternative to conventional antibodies for passive immunization against SARS-CoV-2. They are efficiently produced in prokaryotic expression systems at low cost; exhibit favorable biophysical properties, including high thermostability; and are amenable to engineering of multimeric nanobody constructs with additional benefits (*3*, *32*).

This study identifies four neutralizing nanobodies that target the RBD of SARS-CoV-2 spike from immunized camelids and demonstrates their inhibitory activity in different in vitro infection models. The described set of nanobodies joins a growing list of neutralizing nanobodies (*11*, *12*, *14*–*16*, *18*, *19*, *25*) that have been mostly selected from synthetic libraries or derived from immunizations with related coronaviruses. Without further in vitro evolution, nanobodies isolated from synthetic libraries often exhibit lower affinities than nanobodies selected by the adaptive immune system of immunized animals. Neutralizing nanobodies raised against other coronaviruses are typically not potent toward SARS-CoV-2 in a monovalent form. In this study, we have rationally designed multivalent nanobody constructs on the basis of epitope mapping data by SPR and x-ray crystallography, as well as extensive information on the conformation of spike-nanobody complexes determined by cryo-EM. By using additional functional data on the synergistic behavior of individual nanobody combinations, we developed two classes of nanobody fusions. First, multivalent nanobodies targeting the ACE2 binding site on the RBD (VHH EE and VHH EEE) likely interfere with virus attachment but can be overcome by single point mutations. Second, biparatopic fusions of two nanobodies targeting the nonoverlapping binding interfaces *E* and *UVW* potentiate neutralization by activating SARS-CoV-2 spike. On virions, premature activation likely induces the conversion to the thermodynamically favorable postfusion conformation without catalyzing a fusion event, a process that is irreversible and naturally observed for a fraction of spike trimers on intact SARS-CoV-2 virions (*21*, *33*, *34*). Similar phenomena, albeit mostly observed in biochemical experiments, were proposed for the SARS-CoV-1–NAbs S230 (*35*) and CR3022 (*36*), and MERS-CoV–NAb Mersmab-1 (MERS, Middle East respiratory syndrome) (*37*). Simultaneous binding of VHH VE to the *E* and *UVW* interfaces may involve additional conformational changes not revealed on the unprocessed spike used for EM, possibly facilitating proteolytic processing of S2 to S2′, or bona fide dissociation of S1 from the SARS-CoV-2 spike. Differential sensitivities to protease inhibitors of cell-cell fusion triggered by VHH E and VE warrant more in-depth analysis of the required proteases.

The identification of three nanobodies (VHH E, U, and W)—with two different binding modes targeting independent epitopes that activate the SARS-CoV-2 fusion machinery—suggests that this mode of action may be more common than previously thought. Different coronaviruses employ either the N or the C terminus of the S1 subunit for receptor engagement and activation of fusion by proteinaceous or carbohydrate receptors, suggesting that the spikes of coronaviruses have evolved a pronounced flexibility with regard to activation of the fusion machinery (*38*). It is conceivable that such versatility is achieved by a mode of receptor activation that primarily warrants enhanced susceptibility to secondary proteolytic cleavage to form the S2′ subunit with an exposed fusion peptide, rather than initiating all of the necessary conformational changes required for fusion per se.

The mechanism of fusion activation may have clinical implications, as current models of ADE explain how virus-antibody complexes are taken up by cells expressing Fcγ receptors (*37*, *39*) but fail to explain how the second function of spike-receptor interactions (i.e., the induction of fusion) is achieved. If engagement of the RBD (or S1) by antibodies or nanobodies is sufficient to activate the spike protein, fusion in the absence of cognate receptors, and thus ADE, is more likely to occur. Nanobodies lack the Fc portion and therefore would represent a safe alternative to antibodies, as they cannot be engaged by scavenging receptors to mediate recruitment and uptake of virions into phagocytic cells. The serum half-life of nanobodies can be extended by chemical modification with polyethylene glycol, fusion to human serum albumin (HSA), or fusion to peptides or proteins that bind to HSA with high affinity (*40*).

We predict that bivalent or trivalent nanobody fusions described in this study will also increase the neutralizing potential in vivo. The substantial delay or prevention of escape mutations to biparatopic nanobodies in vitro may translate to better performance in vivo as well. The application of nanobodies by inhalation directly to the site of infection may further reduce the required dose and is also substantially easier to achieve outside of medical facilities (*41*–*43*). Owing to their similarity to the variable domains of the heavy chain of human antibodies, VHHs are generally expected to be less immunogenic than synthetic antibody–like molecules (*44*) and can be further humanized (*45*). In clinical studies of the U.S. Food and Drug Administration–approved nanobody caplacizumab, immunogenicity of nanobodies occurred in 3% of all patients with no effects on clinical efficacy (*46*).

The structure-based multivalent nanobodies presented here have strong potential clinical applications, owing to increased neutralization activity and in-built protection from rapid emergence of escape mutants. We show that premature activation of the spike can be a mechanism for neutralization, and thus the generated nanobodies may also shed more light on the mechanism of fusion itself.

## Materials and methods

### Cell lines

Syrian baby hamster kidney (BHK)-21 cell clone BSR-T7/5 (*Mesocricetus auratus,* RRID:CVCL_RW96, a kind gift of Sean Whelan, Harvard Medical School), human embryonic kidney (HEK) 293T cells (ATCC Cat# CRL-3216, RRID:CVCL_0025), and African green monkey Vero E6 cells (*Chlorocebus sabaeus*, RRID:CVCL_0574, a kind gift of Ralf Bartenschlager, University of Heidelberg) were cultivated in DMEM containing 10% FBS and GlutaMax; human colorectal adenocarcinoma Caco-2 cells (RRID:CVCL_0025) were cultured in DMEM complemented with 10% FBS, 2 mM l-glutamine, 100 μg/ml penicillin/ streptomycin (PS), and 1% nonessential amino acids (NEAA). Flp-In 293 T-REx cells (Thermo Fisher Scientific Cat# R78007, RRID:CVCL_U427) inducibly expressing SARS-CoV-2 spike Δ18 with a C-terminal Strep_2_-HA tag (HEK 293 SARS-CoV-2 spike Δ18) were generated using the Flp-In system (Thermo Fisher Scientific) according to the manufacturer’s recommendations, and cultivated in DMEM supplemented with 10% FBS, Glutamax, 4 μg/ml blasticidin S, and 50 μg/ml hygromycin B. Derivatives of HEK 293 SARS-CoV-2 spike Δ18 cells expressing eGFP or tagRFP-t under the control of CMV promoters were generated using customized gateway-compatible lentiviral vectors based on pRRL (*47*), and cultivated in DMEM supplemented with 10% FBS, Glutamax, 4 μg/ml blasticidin S, 50 μg/ml hygromycin B, and 1 μg/ml puromycin. HEK 293T cells expressing ACE2-tagRFP-t under the control of the EF1α promoter were also generated using a pRRL-based lentiviral vector, and cultivated in DMEM supplemented with 10% FBS, Glutamax, and 50 μg/ml hygromycin B.

### Viruses

All experiments involving the authentic virus were conducted in Biosafety Level 3 laboratories. SARS-CoV-2 clinical isolate SARS-CoV-2/human/Germany/Heinsberg-01/2020 was isolated from a throat swap of an infected patient at the University of Bonn, Germany, and was used for plaque reduction assays (*48*). To prepare larger stocks of virus, Vero E6 cells were infected with SARS-CoV-2 at a multiplicity of infection (MOI) of 0.01. Supernatants were harvested 2 days postinfection (p.i.), cleared by centrifugation, and quantified by plaque assay. To prepare inactivated virus for camelid immunizations, virus was produced in cells covered with Opti-MEM: Clarified supernatants containing virus were mixed with 37% formaldehyde to obtain a final concentration of 4% formaldehyde and incubated at 4°C for 4 hours. The virus suspension was overlaid on 7 ml of a 30% sucrose cushion in 20 mM HEPES pH 7.4, 150 mM NaCl and virions were sedimented by ultracentrifugation in a SW 32 TI rotor at 4°C, 30.000 rpm for 2.5 hours. Inactivated virus pellets from 28 ml of virus-containing supernatants were resuspended in 100 μl of 20 mM HEPES pH 7.4, 150 mM NaCl and the virus suspensions from five ultracentrifuge tubes were pooled. Inactivation of the virus was verified by the lack of replication in a Vero E6 infection experiment. Recombinant SARS-CoV-2 clone icSARS-CoV-2-mNG expressing mNeonGreen (*49*), derived from isolate SARS-CoV-2/human/USA/WA-CDC-WA1/2020, was obtained from the World Reference Center for Emerging Viruses and Arboviruses (WRCEVA) at the UTMB (University of Texas Medical Branch) and was used for microscopy-based replication assays. To generate icSARS-CoV-2-mNG stocks, 200,000 Caco-2 cells were infected with 50 μl of virus stock in a 6-well plate, the supernatant was harvested 48 hours p.i., centrifuged, and stored at −80°C. Viral titers were determined based on mNeonGreen expression after serial dilutions.

To generate replication-deficient pseudotyped VSV strains for single-round infections, we first recovered VSV ΔG eGFP pseudotyped with VSV G, VSV ΔG eGFP (VSV G), from BSR-T7/5 cells using T7-expressing vaccinia virus (VACV) strain vTF7.3, pVSV eGFP dG (a kind gift from Connie Cepko, Harvard Medical School, Addgene plasmid # 31842), and T7-based expression vectors for VSV polymerase (pL), phosphoprotein (pP), nucleoprotein (pN), and glycoprotein (pG) (all kind gifts from Sean Whelan, Harvard Medical School) using published procedures (*50*). VSV ΔG eGFP (VSV G) was amplified in BSR-T7/5 cells transiently transfected with VSV G expression vector pMD2.G (a kind gift from Didier Trono, EPFL, Addgene plasmid # 12259) at 34°C. VSV ΔG eGFP (SARS-CoV-1 SΔ18) and VSV ΔG eGFP (SARS-CoV-2 SΔ18) were produced in HEK 293T cells transiently transfected with pCAGGS SARS-CoV-1 SΔ18 or pCAGGS SARS-CoV-2 S Δ18, respectively, followed by infection with VSV ΔG eGFP (VSV G) and cultivation at 34°C. pCAGGS SARS-CoV-1 S Δ18 encodes amino acids 1 to 1237 of SARS-CoV-1 strain Frankfurt 1 (cloned from a template kindly provided by Stephan Poehlmann, German Primate Center), while pCAGGS SARS-CoV-2 SΔ18 encodes amino acids 1 to 1255 of SARS-CoV-2/human/China/Wuhan-Hu-1/2019 (cloned from a codon-optimized template kindly provided by Stephan Poehlmann). C-terminal truncations were introduced to avoid retention of spike proteins in the ER/Golgi and maximize exposure at the plasma membrane. Virus-containing supernatants were clarified by centrifugation and stored at −80°C. Viral titers were determined by infection of Vero E6 with dilution series of the virus for 8 hours, followed by quantification of green fluorescent cells by flow cytometry. Supernatants were shown to be free of VSV G pseudotyped virus, as no infection of BSR-T7/5 cells was observed.

To generate replication-competent chimeric VSV strains encoding eGFP and SARS-CoV-2 SΔ18 in place of the VSV glycoprotein, we inserted the coding sequence of SARS-CoV-2 SΔ18 (amino acids 1 to 1255 of SARS-CoV-2/human/China/Wuhan-Hu-1/2019) into pVSV eGFP dG. Replication-competent virus was recovered from BSR-T7/5 cells infected with VACV vTF7.3, transfected with pVSV eGFP SARS-CoV-2 SΔ18, pL, pP, pN, and pG, and cultivated at 34°C. Virus was amplified in Vero E6 cells at 34°C and virus-containing supernatants were clarified and stored at −80°C. Viral titers were determined by infection of Vero E6 with dilution series of the virus for 8 hours, followed by quantification of green fluorescent cells by flow cytometry. Chimeric virus strain VSV eGFP SARS-CoV-2 SΔ18 was confirmed to infect primate ACE2 expressing VeroE6 cells, but not BSR-T7/5 cells.

### Proteins

#### Expression and purification of SARS-CoV-2 S RBD

Cloning and expression of the receptor binding domain (RBD) (residues 319 to 541) of spike protein from SARS-CoV-2/human/China/Wuhan-Hu-1/2019 (GenBank: QHD43416.1) was described earlier (*17*). In brief, the coding sequence was cloned into a customized pFastBac vector and fused with an N-terminal gp67 signal peptide and C-terminal His_6_ tag. A recombinant bacmid DNA was generated using the Bac-to-Bac system (Thermo Fisher Scientific). Baculovirus was generated by transfecting Sf9 cells with purified bacmid DNA using FuGENE HD (Promega), and subsequently used to infect suspension cultures of High Five cells (Thermo Fisher Scientific) at an MOI of 5 to 10. Infected High Five cells were cultivated at 28°C for 72 hours, shaking at 110 rpm for protein expression. The supernatant was then concentrated using a 10 kDa MW cutoff Centramate cassette (Pall Corporation). The RBD protein was purified by Ni-NTA, followed by size-exclusion chromatography, and buffer exchanged into 20 mM Tris-HCl pH 7.4 and 150 mM NaCl. Fluorescent RBD was produced by reaction with DyLight 488 NHS ester (Thermo Fisher Scientific) in 100 mM sodium phosphate pH 8.0, 150 mM NaCl, pH 8.0, followed by desalting with 7K MWCO Zeba spin desalting columns (Thermo Fisher Scientific).

#### Expression and purification of nanobodies

Nanobody coding sequences were cloned into pHEN6-based bacterial, periplasmic expression vectors with C-terminal HA-His_6_ or LPETG-His_6_ tags using Gibson cloning (New England Biolabs). Multivalent nanobodies were either directly cloned into pHEN6, or first assembled in pBluescript II (KS) + cloning vectors lacking promoters by Gibson cloning, followed by subcloning into pHEN6 using conventional ligation with T4 ligase. Nanobodies were produced in *E. coli* WK6 transformed with the respective expression vectors. Expression cultures were grown in Terrific Broth (TB), and expression induced with 1 mM IPTG at an OD_600_ of 0.6, followed by cultivation at 30°C for 16 hours. Bacterial pellets were resuspended in TES buffer (200 mM Tris-HCl pH 8.0, 0.65 mM EDTA, 0.5 M sucrose), and periplasmic extracts generated by osmotic shock in 0.25x TES, followed by Ni-NTA purification and either desalting by PD MiniTrap G-25 columns (GE Healthcare Life Sciences) (small scale purifications), or gel filtration with Superdex 75 Increase 10/300 GL or HiLoad 16/600 Superdex 75 pg columns (medium or large scale purifications) in 20 mM HEPES pH 7.4, 150 mM NaCl. Protein was concentrated using Amicon spin-concentrators with 3 or 10 kDa cutoff (Millipore). To produce fluorescently labeled VHHs by sortase A labeling (*51*), 45 μM VHH-LPETG-His_6_ was incubated with 475 μM GGGC-Alexa Fluor 488 and 20 μM His_6_-tagged sortase A 7m for 2 hours. Sortase A 7m and unreacted VHHs were removed by depletion with Ni-NTA, followed by gel filtration on a Superdex 75 Increase 10/300 GL column.

#### Expression and purification of CC12.3 Fab

CC12.3 Fab was produced as described previously (*17*). In brief, the coding sequences of heavy and light chain of CC12.3 Fab were cloned into phCMV3. ExpiCHO cells were transiently co-transfected at a ratio of 2:1 (HC:LC) using ExpiFectamine CHO Reagent (Thermo Fisher Scientific) according to the manufacturer’s instructions. The supernatant was collected at 10 days posttransfection. The Fabs were purified with a CaptureSelect CH1-XL Affinity Matrix (Thermo Fisher Scientific) followed by size-exclusion chromatography.

#### Expression and purification of SARS-CoV-2 spike

Soluble, trimeric SARS-CoV-2 spike was expressed as a fusion of amino acids 1 to 1208 of SARS-CoV-2/human/China/Wuhan-Hu-1/2019 containing mutations R682G, R683S, R685S, K986P, V987P (S2-P), a C-terminal T4 fibritin trimerization motif, an HRV3C protease cleavage site, a TwinStrepTag and a His_8_ tag from a mammalian expression vector based on pαH, as previously described (*6*). For some cryo-EM experiments, the SARS-CoV-2 spike HexaPro mutant with additional proline mutations was used (*52*). In brief, protein was produced in FreeStyle 293F cells transfected with FreeStyle MAX reagent (Thermo Fisher Scientific) or Expi293F cells transfected with ExpiFectamine 293 (Thermo Fisher Scientific). The ectodomain was purified from filtered supernatant on Streptactin XT resin (IBA Lifesciences), followed by gel filtration on a HiLoad 16/600 Superdex 200 column in 50 mM Tris pH 8, 200 mM NaCl. Soluble spike protein was biotinylated using UV-traceable ChromaLink Biotin (SoluLink).

#### Antibodies

The following commercially available antibodies were used: mouse anti-HA antibody HA.11 (clone 16B12, Biolegend, Cat# 901503, RRID:AB_2565005), rabbit anti-DYKDDDDK (FLAG) tag antibody (clone D6W5B, Cell Signaling Technology Cat# 14793, RRID:AB_2572291), HRP-coupled rabbit anti-E tag antibody (Bethyl Laboratories Cat# A190-133P, RRID:AB_345222), HRP-coupled MonoRab rabbit anti-camelid VHH antibody (clone 96A3F5, GenScript Cat# A01860-200, RRID:AB_2734123), HRP-coupled mouse anti-HA-Tag (clone 6E2, Cell Signaling Technology Cat# 2999, RRID:AB_1264166), goat anti-mouse IgG Alexa Fluor Plus 647 (Thermo Fisher Scientific Cat# A32728, RRID:AB_2633277), goat anti-rabbit IgG Alexa Fluor Plus 647 (Thermo Fisher Scientific Cat# A32733, RRID:AB_2633282).

### Nanobody library generation

To elicit heavy-chain–only antibodies against SARS-CoV-2 spike, one 7-year-old male llama (*Lama glama*), and one 6-year-old male alpaca (*Vicugna pacos*) were immunized. All immunizations were authorized by the Landesuntersuchungsamt Rheinland-Pfalz (23 177-07/A 17-20-005 HP). The llama was immunized six times every week with 200 μg SARS-CoV-2 S RBD, complemented with 300 μl formaldehyde-inactivated virus (corresponding to ~10^9^ pfu) in the last two injections. The alpaca was immunized four times every 2 weeks with 200 μg SARS-CoV-2 S RBD. GERBU Adjuvant Fama (GERBU Biotechnik GmbH) was used as an adjuvant in all immunizations. In two (llama) or one (alpaca) injection, respectively, antigen and adjuvant were injected separately. VHH plasmid libraries in the M13 phagemid vector pD (pJSC) were generated as described before (*53*). In brief, RNA from peripheral blood lymphocytes was extracted and used as a template to generate cDNA using three sets of primers (random hexamers, oligo(dT), and primers specific for the constant region of the alpaca heavy chain gene). VHH coding sequences were amplified by PCR using VHH-specific primers, cut with AscI and NotI, and ligated into pJSC linearized with the same restriction enzymes. *E. coli* TG1 cells (Agilent) were electroporated with the ligation reactions and the obtained ampicillin-resistant colonies were harvested, pooled, and stored as glycerol stocks.

### Nanobody identification

SARS-CoV-2 spike RBD-specific VHHs were obtained by phage display and panning with a protocol modified from Schmidt *et al*. (*53*). *E. coli* TG1 cells containing the VHH library were infected with helper phage VCSM13 to produce phages displaying the encoded VHHs as pIII fusion proteins. Phages in the supernatant were purified and concentrated by precipitation. Phages presenting RBD-specific VHHs were enriched using enzymatically or chemically biotinylated RBDs immobilized to MyOne Streptavidin T1 Dynabeads (Thermo Fisher Scientific). The retained phages were used to infect *E. coli* ER2738 and subjected to a second round of panning. 6× 95 *E. coli* ER2837 colonies yielded in the second panning were grown in 96-well plates and VHH expression induced with IPTG. VHHs leaked into the supernatant were tested for specificity using ELISA plates coated with control protein maltose binding protein (MBP) or SARS-CoV-2 spike RBD. Bound VHHs were detected with HRP-coupled rabbit anti-E-Tag antibodies (Bethyl), HRP-coupled MonoRab Rabbit Anti-Camelid VHH Antibody (GenScript), and the chromogenic substrate TMB. Reactions were stopped with 1 M HCl and absorption at 450 nm was recorded. Positive candidates were sequenced, and representative nanobodies cloned into bacterial expression vectors for further analysis.

### ELISA

To test nanobody candidates, SARS-CoV-2 spike RBD or the control protein MBP in PBS were immobilized on ELISA plates at a concentration of 1 μg/mL overnight. Serial 10-fold dilutions of HA-tagged nanobodies in 10% FBS/PBS were incubated with the immobilized antigen, followed by incubation with HRP-coupled anti-HA (clone 6E2, 1:5000, Cell Signaling), and the chromogenic substrate TMB. Reactions were stopped with 1 M HCl and absorption measured at 450 nm.

### Surface plasmon resonance

Surface plasmon resonance experiments were performed using a Biacore 8K instrument (GE Healthcare). The flow system was cleaned using the maintenance “Desorb” function (Desorb Kit, GE Healthcare). The system was flushed with running buffer (20 mM HEPES pH 7.4, 150 mM NaCl, 0.05% Tween 20) and all steps were performed at 25°C chip temperature. Before immobilization, a streptavidin-functionalized sensor chip (Series S Sensor Chip SA, GE Healthcare) was conditioned with three consecutive 1-min injections of 1 M NaCl in 50 mM NaOH (10 μl/min). After immobilization of biotinylated SARS-CoV-2 spike RBD (50 nM, 5 μl/min, 300 s) on the sensor chip flow cell 2, the flow system was washed using 50% isopropanol in 1 M NaCl and 50 mM NaOH. For kinetic binding measurements, various VHHs were injected (30 μl/min, association: 90 s, dissociation: 180 s) over both flows. For epitope binning, the VHHs were pairwise tested for competitive binding using the ABA-injection feature. After each cycle, the surfaces were regenerated with a 120 s injection step of 10 mM glycine pH 2.1. Data were collected at a rate of 10 Hz. The binding data were double referenced by blank cycle and reference flow cell subtraction. Processed data were fitted by the 1:1 interaction model using Biacore Insight Evaluation Software (version 2.0. 15.12933).

### Crystallization and structural determination

VHH E/VHH U/RBD, VHH V/CC12.3/RBD, and VHH W/CC12.3/RBD complexes were formed by mixing each of the protein components at an equimolar ratio and incubating overnight at 4°C. The final concentration for the complexes for crystallization screening ranged from 13.5 to 17.8 mg/ml. Crystallization screening was performed using the vapor-diffusion method in sitting drops containing 0.1 μl of protein and 0.1 μl of reservoir solution with the 384 conditions of the JCSG Core Suite (Qiagen) using the robotic CrystalMation system (Rigaku) at The Scripps Research Institute. Diffraction-quality crystals were obtained in the following conditions

VHH E/VHH U/RBD complex (13.5 mg/ml): 20% PEG 3000, 0.1 M citrate pH 5.5 at 20°C,

VHH V/CC12.3/RBD complex (17.8 mg/ml): 20% PEG 3350, 0.2 M Na2HPO4, pH 9.1 at 20°C,

VHH W/CC12.3/RBD complex (17.6 mg/ml): 1.0 M Li-chloride, 10% PEG 6000, 0.1 M Bicine pH 9.0 at 20°C.

All crystals appeared within 7 days after tray set-up. Before flash cooling in liquid nitrogen for x-ray diffraction studies, crystals were equilibrated in reservoir solution supplemented with 10% ethylene glycol at day 7. Diffraction data were collected at cryogenic temperature (100 K) at either Stanford Synchrotron Radiation Lightsource (SSRL) on beamline 12-1 with a beam wavelength of 0.97946 Å, or at the Advanced Photon Source (APS) at Argonne National Labs on beamline 23ID-B at a wavelength of 1.03317 Å, and processed with HKL2000 (*54*). Structures were solved by molecular replacement (MR) using PHASER (*55*) with MR templates derived from PDB: 6XC7 (*17*), 7JMW (*56*) and 6WAQ (*16*). Iterative model building and refinement were carried out in COOT (*57*) and PHENIX (*58*), respectively. Epitope and paratope residues, as well as their interactions, were identified by accessing PISA at the European Bioinformatics Institute (www.ebi.ac.uk/pdbe/prot_int/pistart.html) (*59*).

### Cryo-EM sample preparation and imaging

Spike trimer (2.4 mg/ml S2-P (*6*) for VHH E and VHH V; HexaPro (*52*) for VHH VE) and nanobody were mixed in a 1:6 molar ratio for VHH E and VHH V, and 1:4 for VHH VE, followed by incubation on ice for 10 min. Prior to cryo-EM grid preparation, grids were glow-discharged with 25 mA for 2 min using an EMS 100X (Electron Microscopy Sciences) glow-discharge unit. Grids used were UltrAuFoil Gold 200 mesh (R 2/2 geometry; Quantifoil Micro Tools GmbH; VHH VE) or CryoMatrix holey grids with amorphous alloy film (R 2/1 geometry; Zhenjiang Lehua Technology Co., Ltd; VHH E, VHH V). 3-μl aliquots of sample solutions were applied to the grids and the grids with sample were then vitrified in a Vitrobot Mk IV (Thermo Fisher Scientific) at 4°C and 100% humidity [blot 10 s, blot force 3, 595 filter paper (Ted Pella Inc.)]. Cryo-EM data collection was performed with EPU (Thermo Fisher Scientific) using a Krios G3i transmission-electron microscope (Thermo Fisher Scientific) operated at 300 kV in the Karolinska Institutet’s 3D-EM facility. Movies were acquired in nanoprobe EFTEM SA mode with a slit width of 10 eV using a K3 Bioquantum for 1.5 s during which 60 movie frames were collected with a fluency of 0.81 e^−^/Å^2^ per frame (see table S3). Motion correction, CTF-estimation, Fourier cropping (to 1.02 Å per pixel), picking and extraction in 600 pixel boxes (size threshold 200 Å, distance threshold 100 Å, using the pretrained BoxNet2Mask_20180918 model) were performed on the fly using Warp (*60*).

A total of 6,468 (VHH E), 9,861 (VHH V), and 12,606 (VHH VE) micrographs were selected based on an estimated resolution cutoff of 4 Å and defocus below 2 microns. and 245,000 (VHH E), 509,302 (VHH V), and 982,221 (VHH VE), particles were picked by Warp (*60*). Extracted particles were imported into cryoSPARC v2.15.0 (*61*) for 2D classification, 3D classification and nonuniform 3D refinement. The particles were processed with C1 symmetry. After 2D classification, clean classes with high-resolution features (and with characteristic trimeric spike views) were retained and used to build ab initio 3D reconstructions. These were further processed for heterogeneous refinement (4.78 Å per pixel) that resulted in reconstructions showing high-resolution structural features in the core of the spike. One round of homogeneous refinement was followed by nonuniform refinement. For the VHH VE dataset, the particles from refinement job containing angular information was migrated from cryoSPARC to RELION 3.1 (*62*) for 3D classification without alignment (1.02 Å per pixel, 35 iterations, *T* = 8) and classified into four classes using reconstruction in cryoSPARC as reference map (low-pass filtered to 25Å). One class containing 92,938 particles, where densities for all six nanobodies were visible, was transferred to cryoSPARC for final refinement in C1 (1.02 Å per pixel). All final reconstructions were analyzed using 3D-FSC (*63*) (figs. S4, S8, and S17) and there was no significant anisotropy in the full map reconstructions (sphericity 0.91 to 0.96). All CTF refinements were local CTF refinements interspersed with global aberration correction (beamtilt, trefoil, tetrafoil and spherical aberration). Please see table S3 for data collection and processing statistics and the respective cryo-EM data processing schemes in figs. S3, S7, and S16. VHH VE and VHH E were pseudo-C3 symmetric especially in the RBD-VHH parts. For VHH E, we symmetry-expanded a particle set with partial-signal subtraction of all parts except for one VHH E-RBD. From this symmetry expanded particle set, we performed local reconstruction of the single VHH E-RBD component. This process significantly enhanced the resolvability of the map and thereby enabled molecular fitting of the density. The locally reconstructed density for RBD-VHH E was then combined with the main map using the combined focused map procedure for refinement as implemented in PHENIX (*58*). A similar procedure was used for local reconstruction of the RBD + VHH VE part from the Spike VHH VE particles and ensuing development of a combined focused map for refinement.

### Cryo-EM model building and structure refinement

The structure of the spike protein trimer PDB: 6ZXN (*15*) was used as a starting model for model building. The structure of the VHHs bound to the RBDs were modeled from the basis of their respective crystallographic models. Structure refinement and manual model building were performed using COOT (*57*) and PHENIX (*58*) in interspersed cycles with secondary structure, Ramachandran, rotamers and bond geometry restrains. Structure figures and EM density-map figures were generated with UCSF ChimeraX (*64*) and COOT (*57*), respectively. Please see table S4 for refinement and validation.

### ACE2-RBD binding assay

HEK 293T cells stably expressing ACE2-tagRFP-t were detached with 1 mM EDTA in PBS. 0.8 μM DyLight 488-labeled SARS-CoV-2 RBD was incubated with different concentrations of VHHs at room temperature (RT) for 60 min. ACE2-expressing cells were subsequently incubated with RBD-VHH mixtures on ice for 30 min. The DyLight 488 signal was measured in all ACE2-tagRFP-t positive cells using a MACS Quant VYB flow cytometer.

### VHH-mediated SARS-CoV-2 spike fusion assay (live-cell imaging)

HEK 293 SARS-CoV-2 spike Δ18 eGFP and HEK 293 SARS-CoV-2 spike Δ18 tag-RFP-t cells were seeded in tissue culture-treated CellCarrier-96 Ultra Microplates (Perkin Elmer) at a density of 100,000 cells of each cell line per well in phenol red-free full medium containing 1 μg/ml doxycycline. 24 hours postseeding, cells were treated with the indicated concentrations of nanobodies and cultivated at 37°C, 5% CO_2_. Every 20 min, images were recorded in four fields of view per well for 14 hours using a Zeiss Observer Z1 wide-field microscope with 20X PlanApochromat objective (NA = 0.8). eGFP and tagRFP-t colocalization was quantified using ImageJ plugin EzColocalization (*65*). Pearson correlation coefficients (PCC) between both channels were determined for each time point, including all pixels (no thresholding).

### SARS-CoV-2 spike-ACE2 fusion assay (live-cell imaging)

HEK 293 SARS-CoV-2 spike Δ18 eGFP and HEK 293T ACE2-tagRFP-t labeled with CellTracker Orange CMRA (1:5000) were seeded in tissue culture-treated CellCarrier-96 Ultra Microplates (Perkin Elmer) at a density of 100,000 cells of each cell line per well in phenol red-free full medium. 24 hours postseeding, cells were treated with 1 μg/ml doxycycline and 1 μM of the indicated nanobodies, and cultivated at 37°C, 5% CO_2_. Every 20 min, images were recorded in four fields of view per well for 14 hours using a Zeiss Observer Z1 wide-field microscope with 20X PlanApochromat objective (NA = 0.8). eGFP and tagRFP-t colocalization was quantified using the ImageJ plugin EzColocalization (*65*). Pearson correlation coefficients (PCC) between both channels were determined for each time point, including all pixels (no thresholding). To normalize fusion, PCC values at 1 hour were subtracted from PCC values of each time point. Average values of four fields of view were corrected by average values of uninduced cells from the same time point. Fusion at *t* = 12 hours was defined as 100% fusion.

### Neutralization assay with VSV ΔG eGFP (SARS-CoV-1/2) (infection assay)

Single-round infection experiments with replication-deficient VSV ΔG eGFP (SARS-CoV-1) and VSV ΔG eGFP (SARS-CoV-2) were performed in Vero E6 cells. 48-well plates were seeded with 3.5·10^4^ Vero E6 cells per well. On the next day, 10^4^ infectious units (IU) of VSV ΔG eGFP (SARS-CoV-1/2) in 50 μl DMEM (without FBS) (titered to achieve ~30% infection) were mixed with 50 μl VHH dilution in DMEM (without FBS), yielding the indicated final VHH concentrations. Virus mixtures were incubated at 37°C for 1 hour, and subsequently used to infect Vero E6 cells. The inoculum was removed after 1 hour and cells were covered with 0.5 ml full DMEM (FBS). After 7 hours at 37°C (8 hours p.i.), cells were trypsinized, fixed, and eGFP-positive cells quantified using a MACS Quant VYB flow cytometer. IC_50_ values were calculated using the “log(inhibitor) vs. normalized response” equation in GraphPad Prism.

### Neutralization assays with SARS-CoV-2 WT (plaque reduction neutralization test)

Plaque reduction neutralization tests (PRNTs) with SARS-CoV-2/human/Germany/Heinsberg-01/2020 were performed in Vero E6 cells. 24-well plates were seeded with 1.5·10^5^ Vero E6 cells per well. The following day, nanobodies were subjected to a two-fold dilution series. 120 μl of each nanobody dilution was mixed with 120 μl of OptiPRO SFM cell culture media (Thermo Fisher Scientific) containing 80 pfu of SARS-CoV-2. After 1 hour at 37°C, 200 μl of each mixture was added to 24-well plates. After 1 hour at 37°C, the inoculum was removed, and cells were overlaid with a 1:1 mixture of 1.5% carboxymethylcellulose (Sigma Aldrich) in 2x MEM (Biochrom) with 4% FBS (Thermo Fisher Scientific). After a 3-day incubation at 37°C, cells were fixed with 6% formaldehyde and stained with 1% crystal violet in 20% ethanol. Plaques were counted manually. IC_50_ values were calculated using the “log(inhibitor) vs. normalized response” equation in GraphPad Prism.

### Neutralization assays with SARS-CoV-2 mNeonGreen (replication assay)

Microscopy-based neutralization experiments with SARS-CoV-2 clone icSARS-CoV-2-mNG were performed with Caco-2 cells. 10^4^ cells/well were seeded in 96-well plates. The next day, cells were infected with icSARS-CoV-2-mNG at an MOI of 1.1 in media containing 5% FBS and serial dilutions of the indicated nanobodies. Cells were subsequently cultivated for 48 hours in the presence of the nanobodies, fixed with 2% PFA, and stained with 1 μg/ml Hoechst 33342 at 37°C for 10 min. The staining solution was removed and exchanged to PBS. To quantify infection rates, images were recorded with a Cytation3 instrument (Biotek). Total (Hoechst positive) and infected (mNeonGreen positive) cells were quantified using the Gen5 Software (Biotek). IC_50_ values were calculated as the half-maximal inhibitory dose using four-parameter nonlinear regression (GraphPad Prism).

### Identification and isolation of escape mutants

To test the emergence or presence of escape mutants of replication-competent VSV SARS-CoV-2 SΔ18 eGFP in the presence of nanobodies, virus was replicated in the presence of serial dilutions of nanobodies in Vero E6 cells (*31*). 1.4·10^5^ cells per well were seeded into 12-well plates. On the next day, 0.5 ml virus dilution (equivalent to an MOI of 0.5) in DMEM (3% FBS, PS) was incubated with 0.5 ml VHH dilution in DMEM (3% FBS, PS) at room temperature for 30 min. The mixture was subsequently transferred to 12-wells with Vero E6 cells and cultivated at 37°C for 4 days. The fraction of eGFP-positive cells as well as cytopathic effect (CPE) were evaluated and selected wells harvested for further cultivation: Cells were lysed in RLT PLUS buffer containing 1% β-mercaptoethanol, followed by purification of RNA with the RNeasy Mini Kit (Qiagen). Supernatants were cleared and stored at 4°C. 1:5 dilutions in DMEM (3% FBS, PS) were used to infect cells for a second round of selection under otherwise identical conditions. eGFP-positive cells and CPE were evaluated 5 days postinfection and selected supernatants and cell lysates harvested.

To quantify escape variants of virus replicated in the presence of neutralizing nanobodies, viral RNA was reversely transcribed to cDNA using a SARS-CoV-2 spike specific primer (FS1957 – 5′-ACTGCTGGAATGGCAGGAAC-3′). The RBD coding sequence flanked by additional 70 bp was amplified by PCR using Phusion DNA polymerase (New England Biolabs) and primer FS1956 (5′-TCTGAGCGAGACAAAGTGCACC-3′) and FS1957, and subjected to Tn5-mediated tagmentation and incorporation of barcoded adapters. Amplicons were sequenced on an Illumina MiSeq machine (v2 nano, 2x150 bp paired-end). After sequencing, FASTQ files were examined using FastQC (Version 0.11.9) and multiqc (Version 1.9). For the alignment, the SARS-CoV-2 spike RBD reference was indexed using bowtie2-build (Version 2.4.1) and samtools (Version 1.10). The reads where aligned using bowtie2 (Version 2.4.1). Conversion into BAM file, sorting and indexing was performed using samtools. BAM files were examined with QualiMap (Version 2.2.2-dev) and multiqc. Variant calling was performed using default GATK HaplotypeCaller (Version 4.1.8.1), and variants were inspected in bam.files using IGV. To quantify the frequencies of mutated interfaces, we determined which mutations within one sample represented individual haplotypes, i.e., in which cases none of the individual reads contained multiple of the mutations (marked with asterisks in tables S5 and S6). Where a global statement was not possible due to the distance of the mutations (marked with hash symbols in tables S5 and S6), the fraction of sequences with at least one mutation was estimated based on visual inspection of individual reads.

To isolate defined escape mutants, resistant virus was amplified from individual virus plaques grown on Vero E6 cells. 1.4·10^5^ Vero E6 cells per well were seeded into 12-wells. The next day, 10-fold serial dilutions of supernatants from virus replicated in the presence of VHH E, U, V, W, E and U, E and V, E and W, or LaM-4 (round 1) were prepared and incubated in the presence or absence of 1 μM of VHHs at RT for 30 min. Vero E6 cells were infected for 30 min at 37°C, followed by overlay of cells with 0.75% agarose, MEM, 2% FBS, PS, 25 mM HEPES with or without the respective VHHs. Two days postinfection, fluorescent plaques grown in the presence of individual nanobodies were identified and marked. No VHH-resistant fluorescent plaques emerged from supernatants of the combinations VHH E and U, E and V, or E and W. A plug of the agarose on top of marked plaques was removed with a Pasteur pipette and incubated in 500 μl DMEM for 4 hours at 4°C. 48-wells were infected with 250 μl of this virus dilution and cultivated in DMEM (2% FBS, PS, 25 mM HEPES) at 37°C until all cells were eGFP-positive. Supernatants were stored at 4°C and cells lysed in RLT PLUS buffer containing 1% β-mercaptoethanol, followed by purification of RNA with the RNeasy Mini Kit (Qiagen). Viral RNA was reversely transcribed to cDNA using a SARS-CoV-2 spike specific primer. The RBD coding sequence flanked by additional 70 bp was amplified by PCR and sequenced by Sanger sequencing. 6/6 plaques resistant to VHH E exhibited the point mutation S494P, 5/5 plaques resistant to VHH U and 5/5 plaques resistant to VHH W encoded the point mutant S371P, and 8/8 plaques resistant to VHH V contained the point mutation K378Q. 4/4 plaques from virus cultivated in the presence of control VHH LaM-4 did not contain any mutations. Selected clones were amplified in Vero E6 cells and cleared supernatants stored at −80°C.

### Growth curves of VSV eGFP SARS-CoV-2 SΔ18 (replication assay)

Vero E6 cells were seeded in 24-well plates (7·10^4^ cells per well). The next day, cells were infected with plaque-purified VSV eGFP SARS-CoV-2 Δ18 isolates at an MOI of 0.02 for 1 hour. The inoculum was removed, cells were washed with PBS twice, and subsequently cultivated in DMEM (2% FBS, 30 mM HEPES, PS). At the indicated time points, supernatants were cleared by centrifugation at 1000 ×g, 4°C, for 10 min and frozen at −80°C until titration. For titration, Vero E6 cells were seeded into 96-well plates at a density of 10^4^ cells per well. Cells were infected with twofold serial dilutions of virus-containing supernatants for 1 hour, followed by cultivation in full medium for 7 hours. Cells were trypsinized, fixed in formaldehyde, and green fluorescence was quantified using a MACS Quant VYB flow cytometer.

## Supplementary Materials

science.sciencemag.org/content/371/6530/eabe6230/suppl/DC1 Supplementary Text Figs. S1 to S23 Tables S1 to S8 References (*68*–*74*) MDAR Reproducibility Checklist Movies S1 to S22

## Appendix

View/request a protocol for this paper from *Bio-protocol*.
